# *Aspergillus violaceofuscus* alleviates cadmium and chromium stress in Okra through biochemical modulation

**DOI:** 10.1371/journal.pone.0273908

**Published:** 2022-10-14

**Authors:** Laila Aziz, Muhammad Hamayun, Mamoona Rauf, Amjad Iqbal, Anwar Husssin, Sumera Afzal Khan, Maryam Shafique, Muhammad Arif, Ayaz Ahmad, Gauhar Rehman, Sajid Ali, Sang Mo Kang, In-Jung Lee

**Affiliations:** 1 Department of Botany, Abdul Wali Khan University Mardan, Mardan, Pakistan; 2 Department of Food Science & Technology, Abdul Wali Khan University Mardan, Mardan, Pakistan; 3 Centre of Biotechnology and Microbiology, University of Peshawar, Peshawar, Pakistan; 4 Department of Microbiology, Federal Urdu University of Art, Science & Technology, Karachi, Pakistan; 5 Department of Biotechnology, Abdul Wali Khan University Mardan, Mardan, Pakistan; 6 Department of Zoology, Abdul Wali Khan University Mardan, Mardan, Pakistan; 7 Department of Horticulture and Life Science, Yeumgnam University, Gyeongsan, Republic of Korea; 8 Department of Applied Bioscience, College of Agriculture and Life Science, Kyungpook National University, Daegu, Republic of Korea; University of Agriculture Faisalabad, PAKISTAN

## Abstract

Endophytic fungi from the Chilli were used to help okra plants exposed to cadmium (Cd) or chromium (Cr) stress. Initially, the strain Ch06 produced higher amounts of indole acetic acid (IAA) (230.5 μg/mL), sugar (130.7 μg/mL), proteins (128.2 μg/mL), phenolics (525.6 μg/mL) and flavonoids (98.4 μg/mL) in Czapek broth supplemented with Cd or Cr. The production of IAA and other metabolites in such a higher concentration suggested that Ch06 might improve plant growth under heavy metal stress. For this reason, an experiment was designed, in which biomass of Ch06 (at 2g/100g of sand) were applied to the okra plants exposed to Cd or Cr stress (at 100 or 500 μg/g). The results exhibited that Ch06 improved the total chlorophyll (36.4±0.2 SPAD), shoot length (22.6±0.2 cm), root length (9.1±0.6 cm), fresh weight (5±0.6 g), dry weight (1.25±0.01 g), sugars (151.6 μg/g), proteins (114.8 μg/g), proline (6.7 μg/g), flavonoids (37.9 μg/g), phenolics (70.7 μg/g), IAA (106.7 μg/g), catalase (0.75 enzyme units/g tissue) and ascorbic acid oxidaze (2.2 enzyme units/g tissue) of the associated okra plants. Similar observations have been recorded in Ch06 associated okra plants under Cd and Cr stress. Also, Ch06 association reduced translocation of Cd (35% and 45%) and Cr (47% and 53%) to the upper parts of the okra plants and thus reduced their toxicity. The internal transcribed spacer (ITS) region amplification of 18S rDNA (ribosomal deoxyribo nucleic acid) exhibited that the potent strain Ch06 was *Aspergillus violaceofuscus*. The results implied that *A*. *violaceofuscus* has the ability to promote host species growth exposed to Cd and Cr. Moreover, it helped the host plants to recover in Cd and Cr polluted soils, hence can be used as biofertilizer.

## Introduction

Soil is the medium that is required as a seedbed for the normal germination of the seed and plant species’ development. Certainly, there are various stresses that can interfere with plant growth [1–4]. Heavy metal (HMs), extreme temperatures, salinity and drought are the main abiotic stressors that can badly affect agriculture [2, 5–7]. Among the abiotic stress factors, the presence of elevated amounts of HMs in the soil is disastrous to the crops [4, 8, 9]. Like all other HMs, higher concentrations of chromium (Cr) and cadmium (Cd) can cause deleterious effect plant species [10]. Excessive Cd in plants may interfere with photosynthesis, enzyme activity, root and shoot lengths and weights and nutrients uptake [11]. Likewise, Cr toxicity may affect nutrients absorption by the crop plants, which in turn affect the plant metabolism that might lead to declination in plant growth [4, 12]. The existence of Cr and Cd in rhizosphere is highly toxic due to its stability and non-biodegradable nature [13]. Therefore, either transformation to stable form or complete elimination of Cr and Cd in the rhizosphere can be vital to protect crops, like okra and achieve sustainable agriculture [13]. Okra is mainly grown in the tropical and subtropical region of Pakistan, which belongs to the Malvaceae family [14]. The crop is easy to grow and has high nutritional, medicinal and commercial value [15]. Though, okra is considered an important vegetable with nutritional and medicinal value, but it is highly susceptible to the stresses. Therefore, the presence of higher amounts of Cr and Cd in soil may cause high losses in their yield during the commercial production.

Phytoremediation of Cd and Cr before okra seed sowing can play a vital role in reclaiming soil, but the process is very time consuming, hence an alternative is needed. Endophytic fungi in this regard have shown remarkable affect in helping the host plants against Cd and Cr stress [9, 10], which is indeed cheap and quick remedy for the crops exposed to stress [9, 10, 16]. Endophytic fungi can live with in host plant species in order to protect them against stresses on the expense of some nutrients [17]. These fungi are known to be omnipresent, multifaceted, and have morphological diversity and greater tolerance to environmental stresses [18]. Such fungi can also support host plant growth in contaminated soils by helping the host to absorb lower quantities of HMs [4, 17], thus making it safe for human consumption [19]. In addition, it can promote host plant by secreting important metabolites, such as flavonoids, phenols, proline, and plant growth promoting phytohormones [20–24]. Intensive research is going on in the field of endophytic fungi, but very little number of species have been reported to help crop plants under stress compared to the species exist in nature. The discovery of small number of endophytic species till date compared to the number of species existed in nature has limited our choice. To have an adequate choice of selecting/using endophytic fungi in crops under Cd and Cr stress conditions, studies need to discover more endophytic fungi with high efficacy. Many studies are conducted in continuous quest to discover highly potent endophytic fungal species that can remediate as well as translocation of the heavy metals to upper parts in order to protect the host crop species and consumers [8, 25]. Keeping the increasing importance of fungal endophytes and demands for food crops in mind, this study was designed with the objectives: (1) Isolation of potent endophytic fungi from chilli plants grown in Cd and Cr contaminated areas. (2) To study the Cd and Cr tolerance of the potent endophytic fungal strain(s) and to monitor the release of indole acetic acid (IAA), phenolics and flavonoids by the endophytes in Czapek broth supplemented with Cd or Cr. (3) To investigate the okra’s response to Cd and Cr in terms of plant growth parameters, proline, enzymatic and non-enzymatic antioxidant systems and leaf chlorophyll concentrations in the presence or absence of potent endophyte(s) isolated from chilli. (4) Identification of the endophytic fungal species with high bioremediation or translocation capacity of Cd and Cr. In short there is sufficient proof to recommend that fungal endophytes can facilitate host plant growth and adaptation to heavy metal stress that can possibly lead to their lucrative application to sustain agriculture.

## Materials and methods

### Endophytes isolation from chilli and further screening

Chilli plants were plugged from various industrial sites in Mardan district of Pakistan with latitude 34.206123° N, and 72.029800° N. The plugged plants were shifted to the zipper bags and stored in the laboratory before further processing. The approach of Hamayun, et al. [20] was pursued to isolate the endophytes from the chilli plants. Initially, leaves, stem, and roots were excised in to small sections (0.5–1 cm) that were sterilized in 70% ethanol. The traces of ethanol from the sterilized pieces were removed by washing them with double distilled water. The sterilized pieces (5 pieces/dish) were then transferred to the petri plates containing Hagem Minimal Medium [agar (1.5%), potassium dihydrogenphosphate (0.05%), glucose (0.5%), magnesium sulphate heptahydrate (0.05%), ferric chloride (0.1%), ammonium chloride (0.05%); pH 5.6±0.2] supplemented with 80 ppm streptomycin and kept for seven days at 27°C. Colorful colonies appeared during the 7-days of incubation period, where each colony represented a single strain of endophytes. Strains from each colony (from parental plates) were repeatedly cultured on PDA (potato dextrose agar) till pure culture obtained. The purified culture of each strain was then shifted to the refrigerator and stored at 4°C. The purified culture from PDA plates was transferred to the conical flask containing Czapek broth (50 mL) with the help of sterilized loop. The spiked broth was then moved to an incubator, ran at 30°C and 120 rpm for 7-days to achieve fungal growth and for the collection of fungal metabolites [26]. Czapek broth (pH 7.3 ± 0.2) consisted of 1% glucose, 1% peptone, 0.05% potassium chloride, 0.001% ferrous sulfate heptahydrate, 0.05% magnesium sulphate heptahydrate). Chilli was used for the isolation of endophytic strains and about 27 fungal strains were preserved. The isolated strains were re-cultured on PDA medium supplemented with cadmium chloride (CdCl_2_) or potassium chromate (K_2_CrO_4_) at 100 or 500 μg/mL concentrations. The susceptible strains were ignored, whereas the successfully grown strains were selected for further experiments.

### Fungal growth rate determination in Cd and Cr supplemented Czapek medium

The Cd and Cr resistant strains (12 in number) were designated as Ch-01 to Ch-12 before employing it in further experiment. Best Cd and Cr resistant strains in heavy metals (HMs) contaminated Czapek broth were selected among the 12 selected endophytic fungal strains. For this purpose, Cd, or Cr in 100 or 500 μg/mL concentrations was exercised. The potency of the endophytes to withstand HMs was noted in Czapek broth, after seven days of incubation in an incubator (120 rpm and 30°C). It was then sieved through a Whatman filter in order to separate the fungal culture filtrate (CF) from the biomass. The collected CF and fresh biomass of the best growing fungi in the Cd or Cr contaminated Czapek broth was kept in the refrigerator (4°C) till use.

### Screening of Cd and Cr resistant strains for growth promotion potential in rice

Growth promotion of the Cd or Cr resistant strains was evaluated initially in rice (*Oryza sativa*) exposed to HMs stress because of their prompt response to the endophytic association, where other crops take some time. Kainat variety of diseased free rice seeds were dipped in ethanol (70%) for 15 mins in order to ensure surface sterilization. The traces of ethanol from the sterilized seeds were removed by showering them with distilled water. The rice seeds were then placed on a two layered damp filter paper in petri plates and allowed to germinate for 3-days. After germination, even sized seedlings (6 seedlings/pot) were selected and moved with great care to the small pots containing 30 mL of 0.8% agar:water medium. The pots were moved to the growth chamber [day: 14 h (28°C±0.3), night 10 h (25°C±0.3), and relative humidity 70%] for further two weeks [27]. When the seedlings reached two-leaf stage, 1 mL of the selected Cd or Cr resistant Ch-06 CF was employed to the apex of the seedlings. The experimental layout was as in Table 1.

**Table 1:** Initial screening of rice seedlings treated with or without Ch-06 CF under Cd and Cr stress.

**Ctrl_DW_**	Rice seedlings irrigated with the distilled water (DW)
**Ctrl_Czpk_**	Rice seedlings irrigated with 5 mL of Czapek broth
**Ctrl_Ch-06_**	Rice seedlings with 5 g of Ch-06 fresh biomass per pot
**Cd1_DW_**	Rice seedlings irrigated with distill water + Cd (100 μg/g sand)
**Cd1_Czpk_**	Rice seedlings with Cd (100 μg/g sand) + 5 mL of Czapek broth per pot
**Cd1_Ch-06_**	Rice seedlings with Cd (100 μg/g sand) + 5 g of Ch-06 biomass per pot
**Cd2_DW_**	Rice seedlings irrigated with distill water + Cd (500 μg/g sand)
**Cd2_Czpk_**	Rice seedlings with Cd (500 μg/g sand) + 5 mL of Czapek broth per pot
**Cd2_Ch-06_**	Rice seedlings with Cd (500 μg/g sand) + 5 g of Ch-06 biomass per pot
**Cr1_DW_**	Rice seedlings irrigated with distill water + Cr (100 μg/g sand)
**Cr1_Czpk_**	Rice seedlings with Cr (100 μg/g sand) + 5 mL of Czapek broth per pot
**Cr1_Ch-06_**	Rice seedlings with Cr (100 μg/g sand) + 5 g of Ch-06 biomass per pot
**Cr2_DW_**	Rice seedlings irrigated with distill water + Cr (500 μg/g sand)
**Cr2_Czpk_**	Rice seedlings with Cr (500 μg/g sand) + 5 mL of Czapek broth per pot
**Cr2_Ch-06_**	Rice seedlings with Cr (500 μg/g sand) + 5 g of Ch-06 biomass per pot

Each treatment was replicated four times. After ten days of the treatment’s application, lengths (shoot and root), and weights (fresh and dry) of the rice seedlings were measured [26].

### Assessment of Cd and Cr in rice and okra seedlings by atomic absorption spectrophotometer (AAS)

AAS (ICE 3000, Thermo Scientific, USA) was used to estimate the endogenous contents of Cd and Cr in rice and okra. Initially, rice or okra samples (0.5 g) were dried (70°C; 72 hs) before acid digestion with nitiric acid (4 mL) and perchloric acid (1 mL). Acid digestion was done at room temperature for 12-h. The acids were evaporated by setting the beakers in a hot water bath for 1 h. When the acids get completely evaporated, the beakers were removed from the water bath and sterilized distilled water (25 mL) was added to re-suspend the contents of the beakers. The contents were then pass through a filter and the filtrate was examined for the quantification of Cd and Cr.

### Selection of potent strains

Among the isolated strains, the Cd and Cr resistant Ch-06 was picked to perform further experiments. The selected strain was enriched and preserved in glycerol stock (50%) at -80°C for future use [28].

### Deoxyribonucleic acid (DNA) extraction

DNA of Ch-06 was obtained by the method of Khan, et al. [29]. Amplification of the Ch-06 DNA was done by PCR. Nano Drop (Thermo Scientific spectrophotometer) at 260 nm was employed to test the quantity and purity of the Ch-06 DNA.

### Ch-06 identification

The 18 S rDNA (ITS region) of Ch-06 was augmented with universal primers, i.e. ITS-1 and ITS-4 [30]. Exactly, 20 ng of Ch-06 genomic deoxy ribo nuclic acid (gDNA) as a template was mixed with 30 μL of reaction mixture (EF-Taq obtained from SolGent, Korea). Activation of Taq polymerase was achieved at 95°C for 2 min; for amplification, 35 cycles at 95°C for a min, and a cycle at 55°C and 72°C for a min each were achieved; for termination, a 10 min step at 72°C was achieved. To visualize the production and estimate the size of PCR product, agarose gel electrophoresis was performed. The same ITS primers were consumed for sequencing the cloned PCR fragment [31]. Code coding aligner (version 7.2.1, Codon code corporations) was consumed to create a consensus sequence through connecting the reverse and forward reads. NCBI was exploited to find the nearest matches in the database after entering the final sequence as a BLAST query (http://www.ncbi.nlm.nih.gov/BLAST). Phylogenetic tree was finally constructed by using the MEGA 7 (version 7.0.18).

### Inoculation of okra with Ch-06 under selected HMs stress

Okra cv. Sabz pari seeds were sterilized in 70% ethanol for 15 mins. The traces of ethanol from the sterilized pieces were removed by washing them with double distilled water. Clean sand free from contamination was autoclaved for 20 mins at 121°C. Seeds (6 seeds/ pot) of okra were then placed in pots containing autoclaved sand (250 g sand/pot) with following treatments (Table 2).

**Table 2:** Okra seedlings treated with or without Ch-06 CF under Cd and Cr stress.

**Ctrl_DW_**	Okra seedlings irrigated with the distilled water (DW)
**Ctrl_Czpk_**	Okra seedlings irrigated with 5 mL of Czapek broth
**Ctrl_Ch-06_**	Okra seedlings with 5 g of Ch-06 fresh biomass per pot
**Cd1_DW_**	Okra seedlings irrigated with distill water + Cd (100 μg/g sand)
**Cd1_Czpk_**	Okra seedlings with Cd (100 μg/g sand) + 5 mL of Czapek broth per pot
**Cd1_Ch-06_**	Okra seedlings with Cd (100 μg/g sand) + 5 g of Ch-06 biomass per pot
**Cd2_DW_**	Okra seedlings irrigated with distill water + Cd (500 μg/g sand)
**Cd2_Czpk_**	Okra seedlings with Cd (500 μg/g sand) + 5 mL of Czapek broth per pot
**Cd2_Ch-06_**	Okra seedlings with Cd (500 μg/g sand) + 5 g of Ch-06 biomass per pot
**Cr1_DW_**	Okra seedlings irrigated with distill water + Cr (100 μg/g sand)
**Cr1_Czpk_**	Okra seedlings with Cr (100 μg/g sand) + 5 mL of Czapek broth per pot
**Cr1_Ch-06_**	Okra seedlings with Cr (100 μg/g sand) + 5 g of Ch-06 biomass per pot
**Cr2_DW_**	Okra seedlings irrigated with distill water + Cr (500 μg/g sand)
**Cr2_Czpk_**	Okra seedlings with Cr (500 μg/g sand) + 5 mL of Czapek broth per pot
**Cr2_Ch-06_**	Okra seedlings with Cr (500 μg/g sand) + 5 g of Ch-06 biomass per pot

Growth chamber [light: 16 h (23°C±1), dark: 8 h (23°C±1)] was employed for the germination okra seeds in pots. After 10 days of okra seed germination, Cd, or Cr was given at 100 μg or 500 μg per g of sand at the interval of 7-days till the harvesting of okra seedlings. The okra seeds were irrigated with distilled water at 3-days interval. Okra seedlings were picked after two months of germination and analyzed for root and shoot lengths (by scale), and fresh and dry weights (by digital balance), total chlorophyll (SPAD), heavy metals, metabolites and antioxidant enzymes.

### Estimation of sugar contents in okra and CF of Ch-06

The modified approach of Mohammadkhani and Heidari [32] was assumed to assess the total soluble sugar contents in fresh leaves of okra and fungal culture filtrate. Fresh leaves (0.5g) samples of okra were ground in mortar and pestle. About 5 mL of double distilled was then added to the pulverized samples and it was centrifuged further for 5 mins at 4,000 rpm. Supernatant (0.1 mL) was taken with great care in a fresh tube and phenol (80%, 1 mL) was added to it. The content of the tube was allowed to stand for 10 min on the bench. Added H_2_SO_4_ (5 mL) to the mixture and allowed to cool for 1 h on the table. The absorbance was finally assessed by a spectrophotometer (Lambda 25 double beam) at 485 nm. The CF of Ch-06 was processed and analyzed in similar way.

### Estimation of proteins contents in okra and CF of Ch-06

Protein contents in okra leaves and CF of Ch-06 was analyzed by the protocol of Lowry, et al. [33]. Reagents were made prior to the experiment; reagent A (to 100 mL of distilled wate added 0.4 g of NaOH, 1 g of KNaC_4_H_4_O_6_.4H_2_O and 2 g of Na_2_CO_3_), reagent B (to 100 mL distilled water added 0.5 g of CuSO_4_.5H_2_O), reagent C (to 50 mL of reagent A added 1 mL of reagent B), reagent D (Mixed equal amounts of distilled water and Folin-phenol reagent). Phosphate buffer (1 mL) was added to the leave samples (1 g) and ground in mortar and pestle. The ground sample was then centrifuged for 10 mins at 3,000 rpm. The collected supernatant (0.1 mL) was supplemented with double distilled water (1 mL) and reagent C (1 mL) was vigorously mixed with the contents of the tube. After mixing, the mixture was supplemented with Reagent D (0.1 mL) and the contents were allowed to stand for 30 min at room temperature. The absorbance was finally assessed at 650 nm. The CF of Ch-06 was processed and analyzed in similar way.

### Assessment of indole acetic acid (IAA) in okra and CF of Ch-06

Salkowski reagent [1 mL of 0.5 M FeCl_3_ (0.5 M) + 50 mL of HClO_4_ (35%)] was used for the assessment of IAA in okra leaves and CF of Ch-06 [34]. For the determination of IAA in okra, 5 mL of double distilled water was added to 0.5 g of the leaves in mortar and pestle before crushing. The crushed sample was centrifuged for 15 mins at 10000 rpm. Salkowski reagent (2 mL) was then added to the supernatant (1 mL) and IAA was finally observed at 540 nm by using spectrophotometer. In terms of Ch-06, Salkowski reagent (2 mL) was added to CF of Ch-06 (1 mL) in a tube and the tube was left to stand for 30 min at ambient temperature in the dark. After incubation the absorbance was recorded with the help of spectrophotometer at 540 nm.

### Assessment of proline in okra and CF of Ch-06

The approach of Bates, et al. [35] was utilized to determine the proline contents in was determined in fresh leaves of okra and CF of Ch-06. Aqueous sulphosalicylic acid (3%; 5 mL) was added to the okra leaves (0.2 g) before crushing. The crushed sample was allowed to cool before centrifugation (at 4,000 rpm for 5 mins) at 5°C for 24 h. To 2 mL of acid ninhydrin reagent (6 M of 20 mL H_3_PO_4_ + 30 mL of CH_3_COOH + 1.25 g of ninhydrin) added 2 mL of okra leaves supernatant or 2 mL of Ch-06 CF. The contents of the tubes were warmed in the water bath for 60 min and 4 mL of toluene was added upon cooling. The absorbance was recorded with the help of spectrophotometer at 520 nm.

### Assessment of flavonoids in okra and CF of Ch-06

Ethanol (80%; 5 mL) was added to fresh leaves of okra (0.5 g) before crushing in mortar and pestle. After crushing, the sample was placed in shaking incubator (120 rpm) for 24 hs. The sample (okra or CF of Ch-06) was then centrifuged (10,000 rpm, 25°C) for 15 mins. From the sample, 0.5 mL of the supernatant was taken in fresh tube and added 0.1 mL of potassium acetate + 0.1 mL of AlCl_3_ (10%) + 4.3 mL of 80% methanol. The contents were vigorously mixed and then incubated for 30 min at room temperature. Absorbance was finally recorded with the help of spectrophotometer at 415 nm [36].

### Assessment of phenolics in CF of Ch-06 and okra

The method of Khatiwora, et al. [36] was used for the determination of total phenolics in CF of Ch-06 and leaves of okra. Methanol (10 mL) was added to leaves sample (0.5 g) before crushing in mortar and pestle. Later water (1 mL) was supplemented with 2 mL of crushed leaves sample or 2 mL of CF. Finally, added 0.5 mL of Folin Cocteau reagent (50%) + 2 mL of Na_2_CO_3_ (20%). The mixture was heated for 1 min and the optical density was recorded upon cooling with the help of spectrophotometer at 650 nm.

### Assessment of catalase (CAT) activity in okra

Phosphate buffer (2 mL) was added to fresh leaves (0.2g) of okra before crushing in mortar and pestle. Centrifugation was done at 10,000 rpm for 5 min and the supernatant was collected and marked as enzyme extract. To the cuvette containing 3 mL of H_2_O_2_-phosphate buffer and placed in spectrophotometer, added 40 μl of enzyme extract. The degradation of H_2_O_2_ by CAT enzyme present in extract was observed at 240 nm. The decrease in 0.05 units of absorbance with time was noted. A decrease of 0.05 units in absorbance at 240 nm were noted as one enzyme unit [37].

### Assessment of ascorbic acid oxidase (AAO) in okra

Phosphate buffer (2 mL) was added to fresh leaves (0.2g) of okra before crushing in mortar and pestle. Centrifugation was done at 4,000 rpm for 5 min and the supernatant was collected and marked as enzyme extract. Substrate solution supplemented with enzyme extract (1 mL) in a cuvette and absorbance was noted at after every 30 sec till 5 min. A decrease of 0.05 units in absorbance were noted as one enzyme unit [38].

### Statistics

The data were collected in replicates and analysed by SPSS software (IBM SPSS Statistics 21) using one way analysis of variance (ANOVA). The significant means were separated through a Duncan’s Multiple Range Test (DMRT) at P = 0.05. Graph Pad Prism (Version 5.03) software was used to construct graphs.

## Results

### Screening of the isolated endophytes from chilli against HMs tress

**Fig 1:**
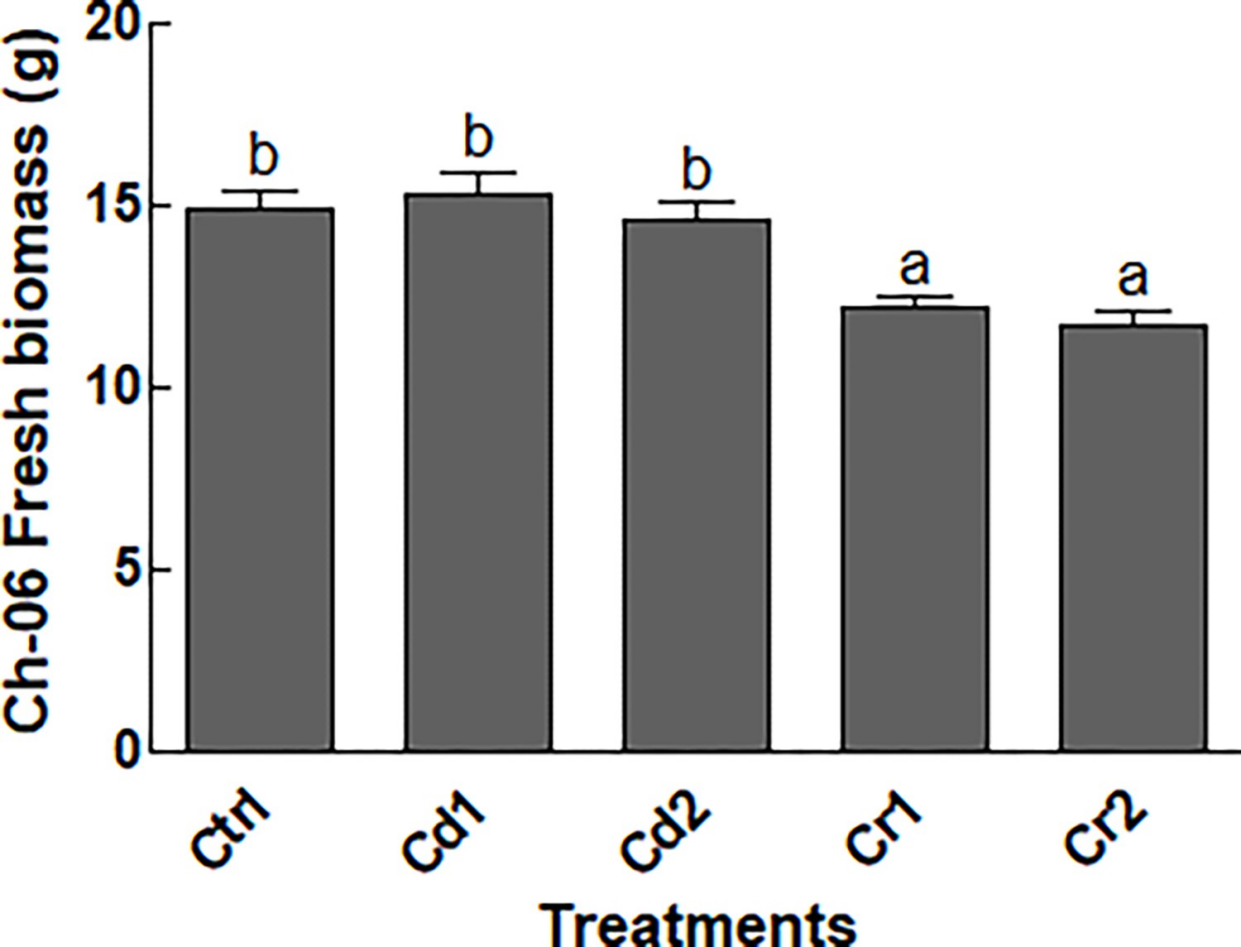
Impact of Cd and Cr stress on biomass Ch-06 fresh biomass. Ctrl = control, Cd = cadmium, Cr = chromium, Cd1 and Cr1 = 100 μg/g, Cd2 and Cr2 = 500 μg/g. The data reflect the mean of triplicated data followed by ±SE. The data with different superscript letter are significantly different at P = 0.05.

The collected *C*. *annum* (chilli) from the industrial areas of Mardan, Pakistan showed the presence of 27 strains. Out of the isolated 27 endophytic fungal strains, 12 have shown growth in PDA medium supplemented with CdCl_2_, K_2_CrO_4_. The selected 12 strains were then exposed to Cd or Cr (at 100 or 500 μg/mL). The results revealed the susceptibility of the two strains (Ch-02 and Ch-08), while the two strains (Ch-03 and Ch-05) showed low growth when exposed to Cd or Cr stress at 500 μg/mL. The notable strain that showed satisfactory growth at higher concentration of Cd or Cr was Ch-06 (results not shown). Furthermore, the fresh biomass of Ch-06 in Czapek media free of HMs after 7-days of incubation was 15.81 g, followed by Cd1 treatment (15.73 g), Cd2 (14.92 g) and Cr1 (14.16 g). Highest decline in the fresh biomass (12.79) of Ch-06 was recorded in Czapek media supplemented with Cr at 500 μg/mL (Fig 1).

### Ch-06 association and rice growth exposed to Cd or Cr stress

The effect of Cd or Cr stress in the presence of Ch-06 was less pronounced on the growth parameters of the rice seedlings. Maximum shoot (18.7 cm) and root lengths (10.7 cm) were achieved by the Ch-06 associated rice seedlings, whereas seedlings treated with Czapek (14.7 and 5.5 cm) and distilled water (13.6 cm, 5.4 cm) achieved lower values. On the contrary, shoot and root lengths of Ch-06 free rice seedlings were significantly (P = 0.05) decreased by Cd or Cr (at 100 or 500 μg/mL) stress in comparison to the Ch-06 associated rice seedlings (Table 3). Likewise, a considerable decrease in shoot and root weights (fresh and dry) were recorded for Ch-06 free rice seedling exposed to Cd or Cr stress. However, Ch-06 inoculation played a vital role in the alleviation of Cd and Cr stress as reflected by the weights of shoot and root (fresh and dry) of rice seedlings (Table 3).

**Table 3:** Effect of Ch-06 on rice under Cd and Cr stress.

Treatment	SL (cm)	RL (cm)	FWg (g)	DWg (g)
**Ctrl**_**DW**_	13.6±0.3^de^	5.4±0.2^de^	0.09±0.001^ef^	0.021±0.001^cd^
**Ctrl**_**Czpk**_	14.7±0.5^e^	5.5±0.2^e^	0.10±0.002^f^	0.022±0.002^d^
**Ctrl**_**Ch-06**_	18.7±0.6^f^	10.7±0.4^h^	0.12±0.002^h^	0.028±0.002^e^
**Cd1**_**DW**_	12.8±0.5^cd^	5.3±0.2^de^	0.09±0.003^de^	0.020±0.001^cd^
**Cd1**_**Czpk**_	13.8±1.0^de^	5.6±0.3^e^	0.91±0.002^ef^	0.021±0.005^cd^
**Cd1**_**Ch-06**_	18.5±0.5^f^	10.9±0.3^h^	0.12±0.002^h^	0.028±0.001^e^
**Cd2**_**DW**_	11.1±0.1^ab^	3.5±0.2^ab^	0.07±0.001^ab^	0.016±0.002^ab^
**Cd2**_**Czpk**_	13.2±0.3^cde^	4.6±0.2^cd^	0.09±0.004^de^	0.020±0.002^cd^
**Cd2**_**Ch-06**_	17.7±0.6^f^	9.1±0.1^g^	0.12±0.002^gh^	0.027±0.001^e^
**Cr1**_**DW**_	11.6±0.5^bc^	4.3±0.2^bc^	0.08±0.001^cd^	0.018±0.001^abcd^
**Cr1**_**Czpk**_	12.3±0.2^bcd^	4.9±0.1^cde^	0.08±0.001^bc^	0.019±0.001^bcd^
**Cr1**_**Ch-06**_	18.0±0.6^f^	10.3±0.6^h^	0.12±0.002^gh^	0.027±0.002^e^
**Cr2**_**DW**_	9.6±0.3^a^	3.2±0.1^a^	0.07±0.003^a^	0.015±0.001^a^
**Cr2**_**Czpk**_	10.9±0.5^ab^	3.7±0.1^ab^	0.07±0.002^ab^	0.017±0.001^abc^
**Cr2**_**Ch-06**_	17.3±0.8^f^	8.2±0.1^f^	0.11±0.002^g^	0.026±0.002^e^

Ctrl = control, Cd = cadmium, Cr = chromium, Cd1 and Cr1 = 100 μg/g, Cd2 and Cr2 = 500 μg/g, DW = distilled water, Czpk = Czapek broth, SL = shoot length, RL = root length, FWg = plant fresh weight, DWg = plant dry weight. The data reflect the mean of triplicated data followed by ±SE. The data with different superscript letter are significantly different at P = 0.05.

### Cd and Cr accumulation in Ch-06 associated rice seedlings

**Fig 2:**
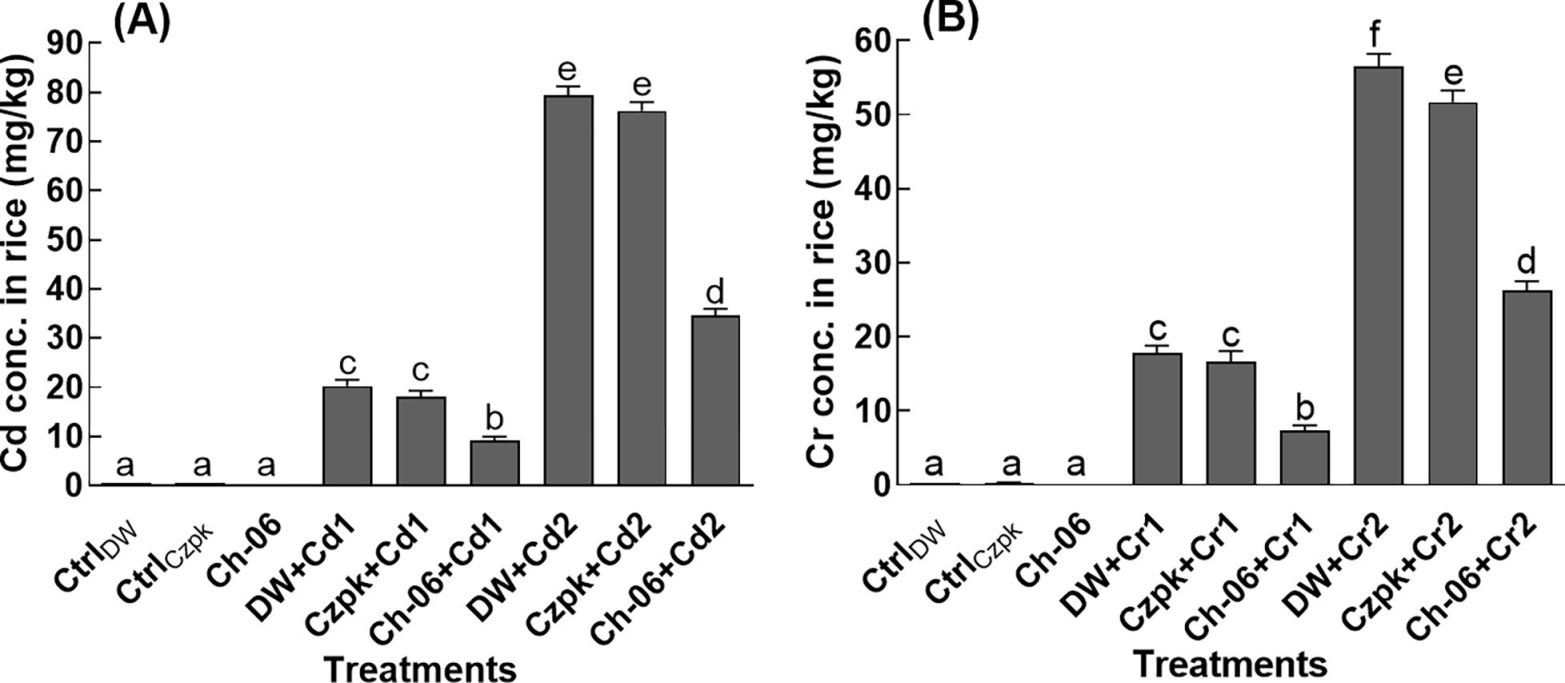
Cd and Cr concentration in Ch-06 associated rice seedlings. Cd = cadmium, Cr = chromium, Cd1 and Cr1 = 100 μg/g, Cd2 and Cr2 = 500 μg/g. Fig A = Cd concentration, Fig B = Cr concentration. The data reflect the mean of triplicated data followed by ±SE. The data with different superscript letter are significantly different at P = 0.05.

The results of Fig 2 displayed a significant (P = 0.05) decline in Cd and Cr accumulation in Ch-06-associated rice seedlings compared to the Ch-06-free seedlings. A 68.9% and 69.68% reduction was recorded for the Cd in the Ch-06-associated rice seedlings compared to the non-associated DW treated/control seedlings. Likewise, a reduction of 70.9% and 68.24% for Cr was found in Ch-06 associated rice seedlings in comparison to the non-associated DW treated/control seedlings (Fig 2).

### Identification of endophyte Ch-06

The outcome of DNA sequence analysis disclosed the strain Ch-06 as an *A*. *violaceofuscus*. The accession number MH577055 was received at the submission of DNA sequence to the NCBI GenBank.

### Presence of the selected primary and secondary metabolites in *A*. *violaceofuscus* CF

**Fig 3:**
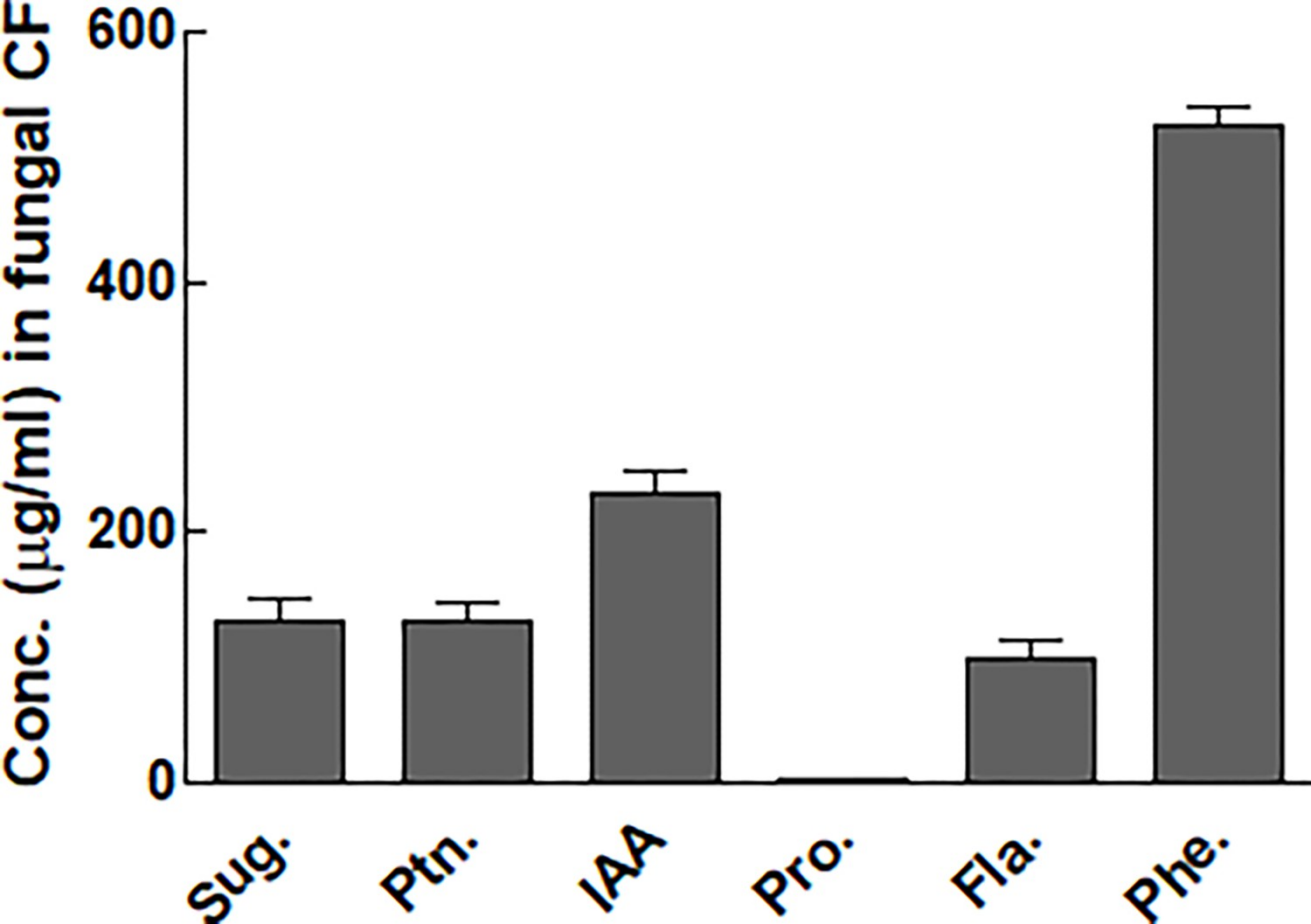
Concentration of total soluble sugar, proteins and secondary metabolites produced by Ch-06 in Czapek broth media. The data reflect the mean of triplicated data followed by ±SE. The data with different superscript letter are significantly different at P = 0.05.

The endophytic fungi, Ch-06 released appreciable amounts of primary and secondary metabolites in their CF (Fig 3). The observed sugar and protein levels in CF of Ch-06 were 130.8 μg/mL and 128.2 μg/mL. Similarly, higher amounts of IAA (230.5 μg/mL), proline (3.5 μg/mL) and flavonoids (98.4 μg/mL) were present the CF of Ch-06 (Fig 3).

### *A*. *violaceofuscus* mitigate Cd and Cr toxicity in okra

The application of the endophytic fungi *A*. *violaceofuscus* biomass to okra seedlings exhibited a significant (P = 0.05) effect on the various growth parameters of okra seedlings under all conditions (Table 4). A significant (P = 0.05) decrease in seedling lengths were attained by the fungi free okra seedlings at 100 or 500 μg/mL of Cd or Cr in comparison to the associated okra plants. Similarly, a substantial decline in shoot and root weights (fresh and dry) were recorded in endophyte free rice seedling exposed to Cd or Cr stress. However, its association with host improved the shoot and root weights (fresh and dry) of the okra seedlings exposed to Cd or Cr stress all tested concentrations (Table 4).

**Table 4:** Effect of Ch-06 on okra under Cd and Cr stress.

Treatment	Total Chl. (SPAD)	SL (cm)	RL (cm)	FWg (g)	DWg (g)
**Ctrl**_**DW**_	34.82±0.12^ab^	15.72±0.20^fgh^	5.54±0.20^cde^	3.48±0.02^bc^	0.87±0.005^cd^
**Ctrl**_**Czpk**_	35.04±6.30^ab^	16.51±0.35^h^	6.71±0.10^fg^	3.66±0.06^cd^	0.92±0.060^d^
**Ctrl**_**Ch-06**_	36.41±0.20^ab^	22.62±0.20^k^	9.11±0.60^i^	5.04±0.60^e^	1.25±0.005^e^
**Cd1**_**DW**_	35.13±0.20^ab^	14.83±0.10^ef^	5.43±0.20^cde^	3.31±0.01^abc^	0.82±0.010^bcd^
**Cd1**_**Czpk**_	34.01±0.98^ab^	15.91±0.70^gh^	6.34±0.20^efg^	3.53±0.50^bc^	0.88±0.060^cd^
**Cd1**_**Ch-06**_	37.22±0.12^b^	21.70±0.10^jk^	8.71±0.10^i^	4.84±0.10^e^	1.22±0.010^e^
**Cd2**_**DW**_	34.73±0.10^ab^	12.81±0.04^bc^	4.11±0.10^ab^	2.80±0.020^abc^	0.71±0.010^abc^
**Cd2**_**Czpk**_	33.04±0.90^ab^	13.74±0.70^cd^	5.12±0.20^bcd^	3.03±0.60^abc^	0.76±0.060^a-d^
**Cd2**_**Ch-06**_	35.91±0.10^ab^	20.81±0.50^ij^	8.10±0.20^hi^	4.63±0.01^e^	1.15±0.010^e^
**Cr1**_**DW**_	34.73±0.40^ab^	14.11±0.20^de^	4.92±0.03^bcd^	3.12±0.10^abc^	0.78±0.001^a-d^
**Cr1**_**Czpk**_	33.61±0.40^ab^	15.13±0.30^efg^	5.81±0.10^def^	3.41±0.03^bc^	0.84±0.003^bcd^
**Cr1**_**Ch-06**_	36.12±0.30^ab^	20.81±0.20^ij^	8.52±0.80^i^	4.61±0.10^e^	1.16±0.030^e^
**Cr2**_**DW**_	31.14±0.70^a^	11.34±0.30^a^	3.42±0.60^a^	2.54±0.02^a^	0.63±0.001^a^
**Cr2**_**Czpk**_	32.53±0.05^ab^	12.32±0.20^ab^	4.52±0.30^bc^	2.71±0.10^ab^	0.68±0.050^ab^
**Cr2**_**Ch-06**_	35.51±0.90^ab^	20.21±0.30^i^	7.21±0.05^gh^	4.34±0.10^de^	1.13±0.200^e^

Ctrl = control, Cd = cadmium, Cr = chromium, Cd1 and Cr1 = 100 μg/g, Cd2 and Cr2 = 500 μg/g, DW = distilled water, Czpk = Czapek broth, SL = shoot length, RL = root length, FWg = plant fresh weight, DWg = plant dry weight. The data reflect the mean of triplicated data followed by ±SE. The data with different superscript letter are significantly different at P = 0.05.

### Cd and Cr accumulation in *A*. *violaceofuscus* treated okra seedlings

**Fig 4:**
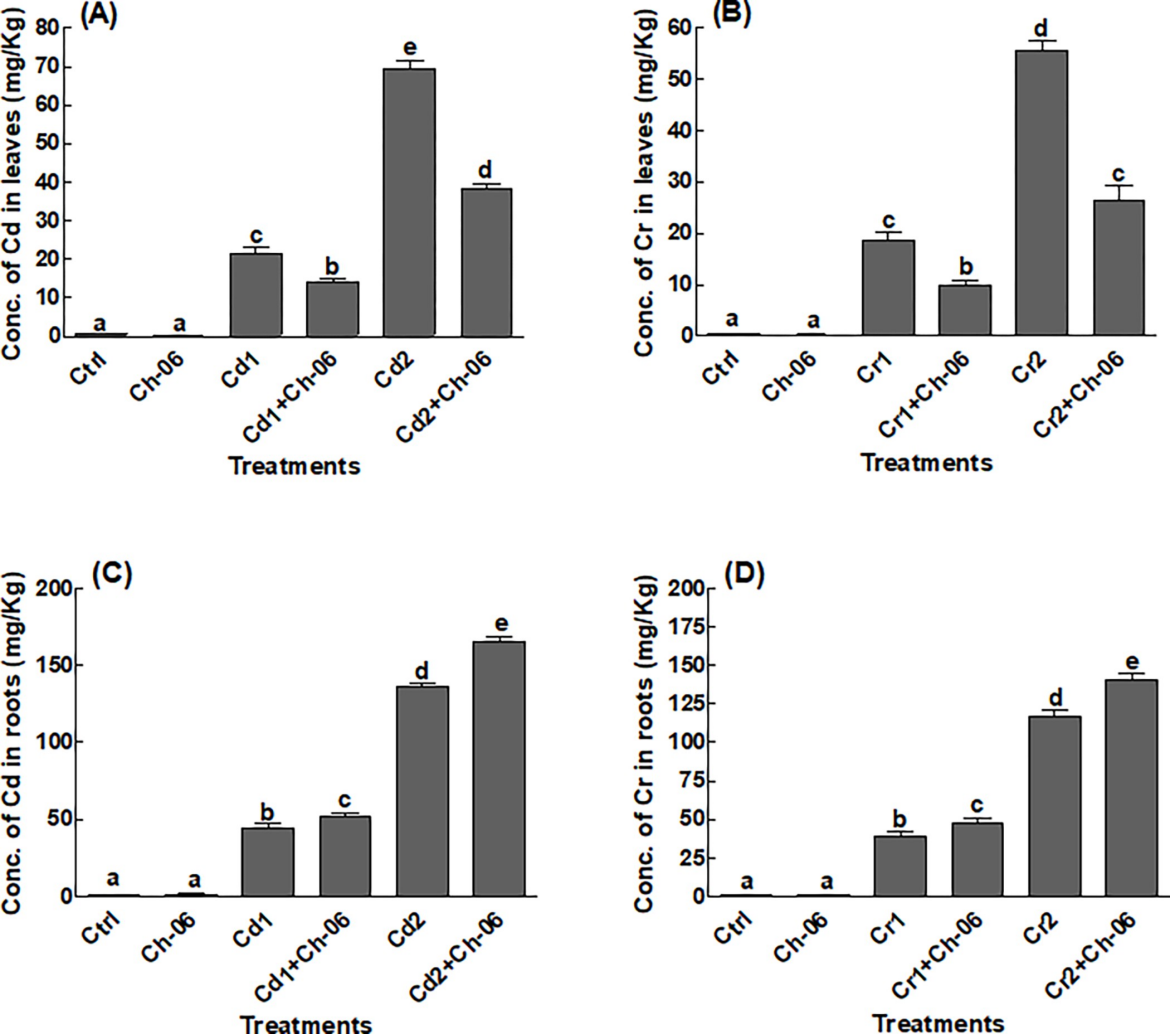
Impact of Ch-06 (*A*. *violaceofuscus*) on endogenous Cd and Cr content under elevated Cd and Cr stress. Cd = cadmium, Cr = chromium, Cd1 and Cr1 = 100 μg/g, Cd2 and Cr2 = 500 μg/g. (A): Cd or Cr concentration in okra leaves; (B): Cd or Cr concentration in okra roots. The data reflect the mean of triplicated data followed by ±SE. The data with different superscript letter are significantly different at P = 0.05.

*A*. *violaceofuscus* significantly (P = 0.05) influenced the building up of Cd or Cr in the leaf and roots of okra seedlings (Fig 4). A significantly lower (P = 0.05) amounts of Cd and Cr was noticed in the endophytic fungi inoculated okra seedlings in comparison to the free seedlings. After exposing the okra to Cd or Cr (at 100 or 500 μg/mL), significant (P = 0.05) quantities of Cd or Cr were accrued in the endophyte free okra leaves in comparison to the associated okra leaves (Fig 4A & 4B). On the contrary, a completely opposite trend was noticed in *A*. *violaceofuscus* free and associated roots. Significant (P = 0.05) amounts of Cd or Cr in the roots of the inoculated okra roots in comparison to the non-associated okra roots (Fig 4C & 4D).

### Impact of *A*. *violaceofuscus* on metabolites of okra

**Fig 5:**
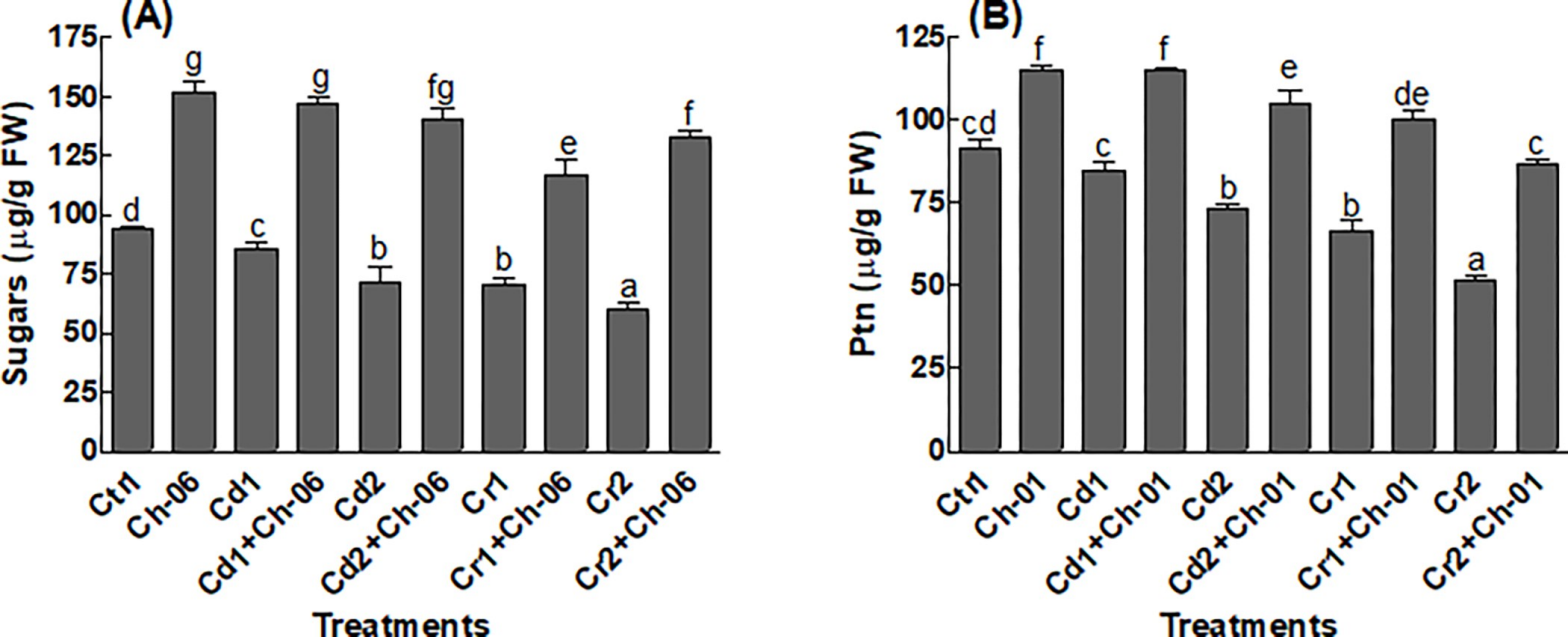
Impact of Ch-06 (*A*. *violaceofuscus*) on soluble sugar and protein contents of okra under elevated Cd and Cr stress. Co: Control, Cd = cadmium, Cr = chromium, Cd1 and Cr1 = 100 μg/g, Cd2 and Cr2 = 500 μg/g. (A): Soluble sugar; (B): Proteins. The data reflect the mean of triplicated data followed by ±SE. The data with different superscript letter are significantly different at P = 0.05.

**Fig 6:**
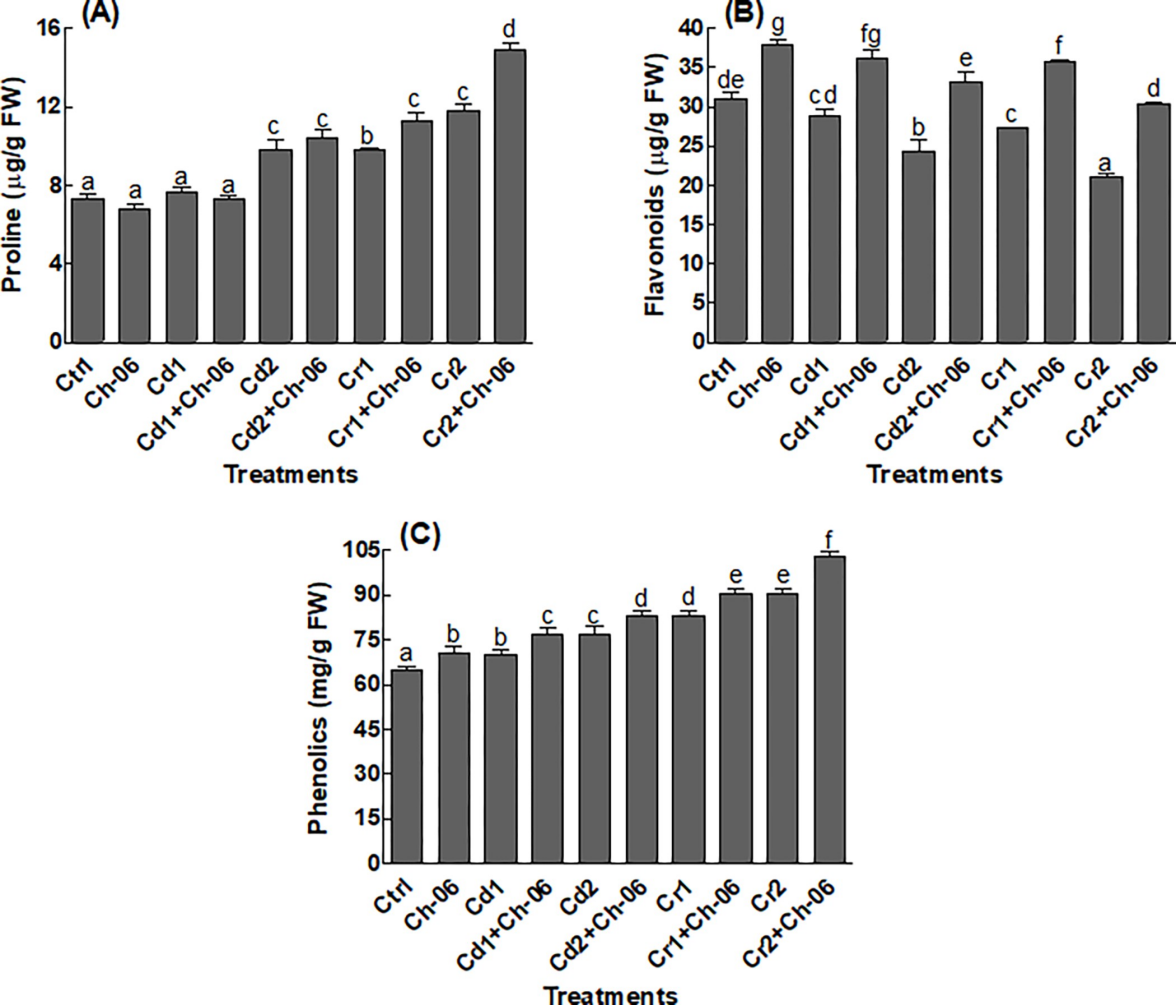
Impact of Ch-06 (*A*. *violaceofuscus*) on IAA content of okra under elevated Cd and Cr stress. Co: Control, Cd = cadmium, Cr = chromium, Cd1 and Cr1 = 100 μg/g, Cd2 and Cr2 = 500 μg/g. The data reflect the mean of triplicated data followed by ±SE. The data with different superscript letter are significantly different at P = 0.05.

Endophytic fungi *A*. *violaceofuscus* associated okra seedlings under all conditions exhibited significant (P = 0.05) amounts of sugars and proteins in comparison to the control plants (Fig 5A & 5B). Furthermore, significant (P = 0.05) reduction in sugars and proteins were noted in the endophyte free seedlings provided with Cd or Cr (Fig 5A & 5B). Similarly, endophytic association significantly (P = 0.05) improved the proline, flavonoid and phenolic contents of okra under all treated conditions (Fig 6). Higher amounts of proline (14.9 μg/g) were found in endophytic fungi associated okra seedlings exposed to Cr even at 500 μg/mL stress (Fig 6A). Likewise, *A*. *violaceofuscus* association promoted the production of flavonoids and phenolics in okra seedlings exposed to Cd or Cr in comparison to the free okra (Fig 6B & 6C).

### Impact of *A*. *violaceofuscus* on okra IAA

**Fig 7:**
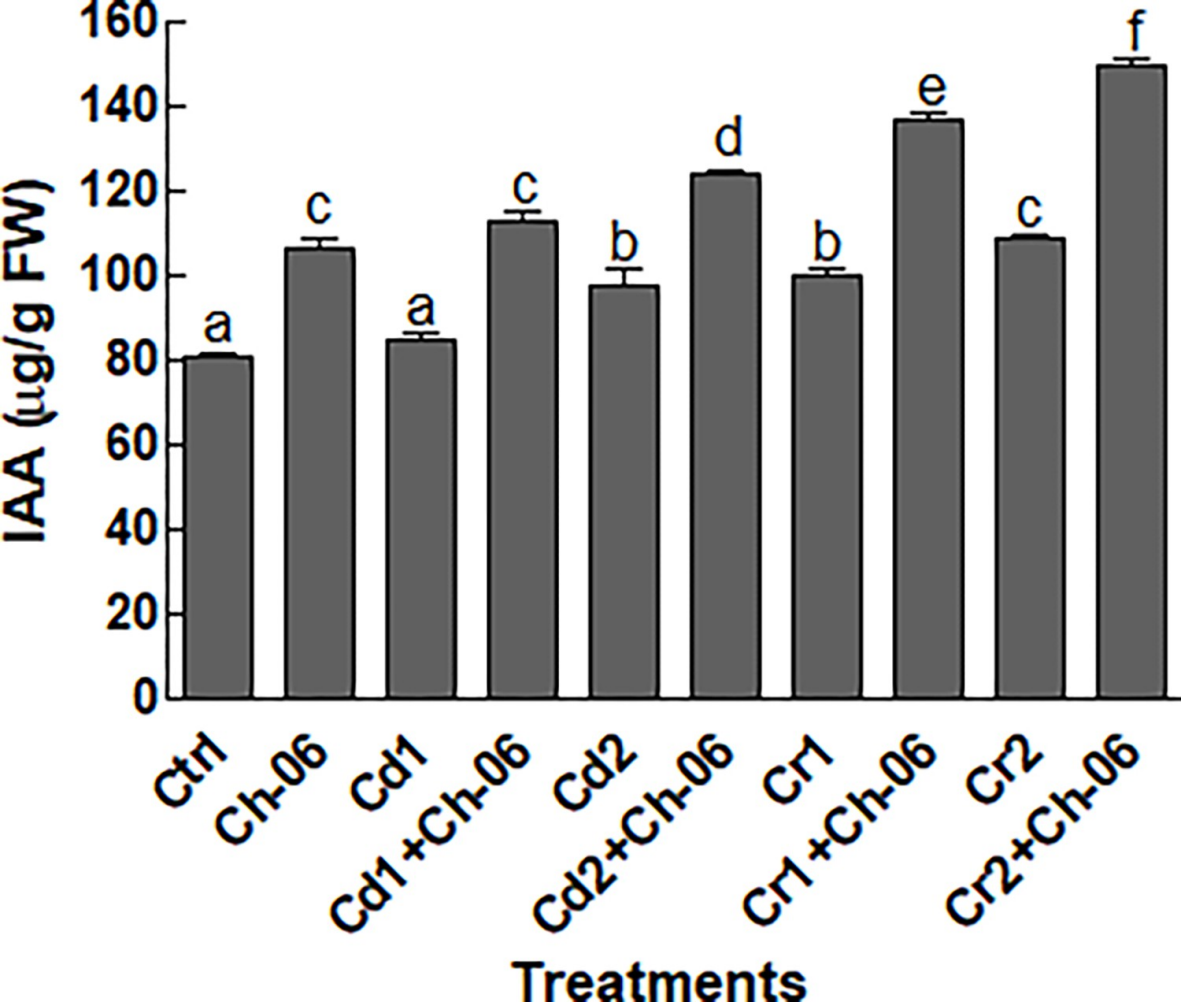
Impact of Ch-06 (*A*. *violaceofuscus*) on proline, flavonoids and phenolic contents of okra under elevated Cd and Cr stress. Co: Control, Cd = cadmium, Cr = chromium, Cd1 and Cr1 = 100 μg/g, Cd2 and Cr2 = 500 μg/g. (A): Proline, (B): Flavonoids, (C): Phenolics. The data reflect the mean of triplicated data followed by ±SE. The data with different superscript letter are significantly different at P = 0.05.

The results in Fig 7 shows that the association of *A*. *violaceofuscus* significantly (P = 0.05) enhanced the IAA levels in okra seedlings subjected to Cd or Cr stress (at 100 or 500 μg/mL). Maximum amounts (150.13 μg/g) of IAA were recorded in endophytic fungi associated okra seedlings exposed to Cr at 500 μg/g (Fig 7).

### Impact of *A*. *violaceofuscus* on CAT and AAO activities of okra

**Fig 8:**
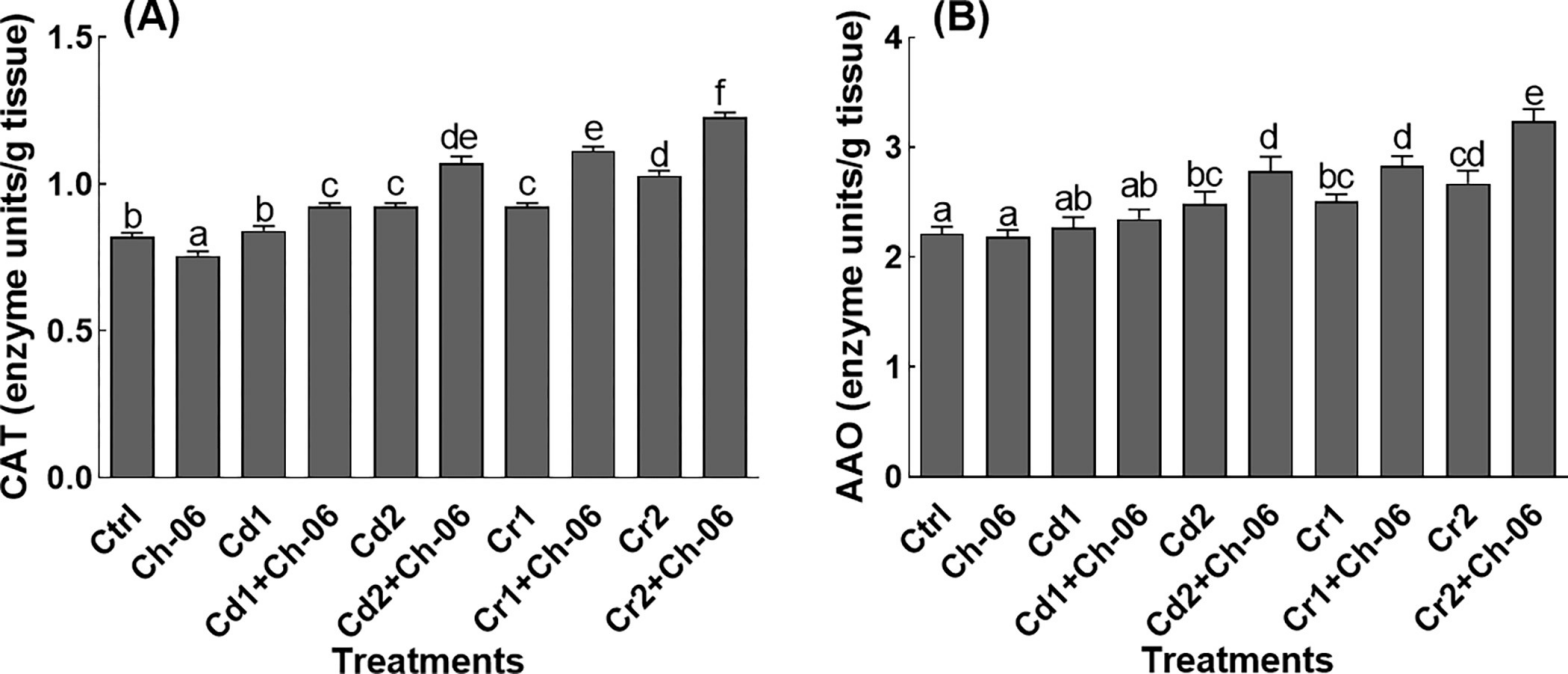
Impact of Ch-06 (*A*. *violaceofuscus*) on CAT and AAO levels of okra under elevated Cd and Cr stress. Co: Control, Cd = cadmium, Cr = chromium, Cd1 and Cr1 = 100 μg/g, Cd2 and Cr2 = 500 μg/g. (A): Catalase, (B): Ascorbic acid oxidase. The data reflect the mean of triplicated data followed by ±SE. The data with different superscript letter are significantly different at P = 0.05.

Endophytic fungi *A*. *violaceofuscus* associated okra seedlings under normal conditions exhibited low activity of CAT and AAO in comparison to the free seedlings (Fig 8A & 8B). Conversely, higher activities of CAT and AAO were recorded in endophyte associated okra seedlings exposed to Cd or Cr (at 100 or 500 μg/mL) stress in comparison to the free seedlings (Fig 8A & 8B).

## Discussion

Earth’s crust naturally consists of various metals including heavy metals (HMs), but exponential growth in domestic, agricultural and advancement in technology have influenced their geochemical cycle and biochemical [39]. The existence of meaningful quantities of these heavy metals in cultivable lands can lead to the accretion of toxic HMs in various parts of the plants. The HMs deposits in cops will not only affect the physiological and biochemical processes of crops, but subsequently causes health issues in animals and humans after eating HMs contaminated crops [19]. As human is getting effected by the toxic levels of HMs necessary steps are required to solve this problem. In the past few decades, the utility of plant species to remove HMs from the soil got great appreciation, but now a day’s soil reclamation through microorganisms is getting more popularity [4]. The use of microbes to help the crop plants in the HMs contaminated soils is considered to be more practical, easy, cost effective and safe to the ecosystem [4, 9, 25]. Exploration of beneficial microbes, such as endophytes (microbes live in symbiotic relations with host plants) and their application in HMs contaminated soils can lead to a sustainable agriculture and access to a safer food [4, 16, 40].

Qadir, et al. [8] observed Cr remediation potential of endophytic *Aspergillus niger* in sunflower. The impact of endophytic fungi *A*. *violaceofuscus* on the physiological and biochemical parameters of okra seedlings exposed to Cd or Cr stress was explored in this study. The endophytic fungi promoted the various growth parameters of okra seedlings exposed to Cd or Cr stress. Certainly, Cd or Cr exposures have negatively influenced the shoot and root lengths, and shoot and root weights (fresh and dry) of okra seedlings. However, fungal association improved the length and weight of the okra plants under Cd or Cr stress. The advancement in various growth parameters of the *A*. *violaceofuscus* associated okra seedlings could possibly be due to exogenous supply of IAA as produced by the endophyte [17]. Similarly, beneficial microorganisms have been observed to release various phytohormones (IAA, GA and SA) to alleviate stress conditions and promoted the growth parameters in the host plant species [4].

Furthermore, the effect of Cd or Cr on okra seedlings were less pronounced in the presence of endophytic fungi *A*. *violaceofuscus*. After analyzing the leaves of the fungal associated and free seedlings exposed to Cd or Cr, we found low buildup of these HMs in the associated okra leaves. This might be due to the ability of the fungal endophyte to evade the translocation of Cd and Cr in to the leaves and other edible parts of the okra plants (Bibi et al., 2018). Also, it could be possible that the endophytic fungi *A*. *violaceofuscus* assisted the host plant species by retaining the Cd and Cr in their mycelia and hindered its translocation to the shoots of host tissues [41]. On the contrary, the results of this study exhibited higher amounts of Cd or Cr in the roots of fungal inoculated okra seedlings in comparison to the non-inoculated seedlings at 100 or 500 μg/mL of Cd or Cr stress. Endophytes have the potential of binding the HMs to its released extracellular polymers, therefore, keeping its host safe from the toxic effect of HMs [16]. Shahabivand, et al. [42] also detected higher quantities of HMs in the roots rather leaves of various crop species inoculated with *P*. *indica*. Since roots interact with the HMs contaminated soil directly, it might be possible that root accrue higher amounts of HMs as compared to the shoots or leaves of the same plant species [43].

Plant releases higher amounts of metabolites during stress conditions to avoid serious damages to their tissues [17]. The results of this study also showed high metabolites production by okra plants under Cd or Cr stress. Additionally, the concentration of the produced metabolites in okra seedlings inoculated with *A*. *violaceofuscus* were higher than the non-inoculated okra exposed to Cd or Cr stress. In past, various scientists have recorded higher production of IAA, flavonoids, phenolics and proline in endophyte associated plant species that helped them to resist stress [4, 16, 44]. Besides the above mentioned metabolites, okra seedlings associated with endophytic fungi produced higher levels of sugars and proteins in comparison to the non-associated seedlings. The amounts of the sugars decreased okra seedlings under stressful conditions could be linked with high respiration and low photosynthesis rates [10]. Likewise, HMs has the ability to destabilize the proteins in plant species exposed to HMs stress, but inoculation of the host plant species with endophytes might prevent the factors that can inhibit the synthesis of proteins [45]. Also, higher activities of CAT and AAO were recorded in *A*. *violaceofuscus* associated okra seedlings exposed to Cd or Cr in comparison to the free seedlings. The results of our study are comparable to the findings of Bilal, et al. [21], who reported that endophytic fungi can encourages the production of CAT and AAO in plant species under HMs stress. The production of CAT and AAO above their optimal levels under stress can help the plant species to lower the quantities of reactive oxygen species and to ease oxidative stress.

## Conclusion

The results concluded that the isolated fungal endophyte *A*. *violaceofuscus* is not only a plant growth promoter, but capable of reducing Cd and Cr toxicity and their adverse effects in associated okra plants compared to the non-inoculated control plants. Moreover, the endophytic fungal association improved the host plant (okra) resistance against Cd or Cr (at 100 or 500 μg/mL) stress by improving physiological (shoot length, root length, fresh weight, dry weight) and biochemical (total chlorophyll, sugars, proteins, proline, flavonoids, phenolics, IAA, catalase and ascorbic acid oxidaze) parameters. The endophytic fungal association also reduced Cd and Cr translocation to the upper parts of the okra plants in order to ease the negative effects. This bio-efficient fungal endophyte could be a promising alternative to phytoremediation in reducing the Cd and Cr stress and improving the health of okra plants. However, for future prospects, it can be helpful to use *A*. *violaceofuscus* as a potential biofertilizer in crops cultivated in Cd or/and Cr contaminated soils.
